# Early Presymptomatic and Long-Term Changes of Rest Activity Cycles and Cognitive Behavior in a MPTP-Monkey Model of Parkinson's Disease

**DOI:** 10.1371/journal.pone.0023952

**Published:** 2011-08-24

**Authors:** Julien Vezoli, Karim Fifel, Vincent Leviel, Colette Dehay, Henry Kennedy, Howard M. Cooper, Claude Gronfier, Emmanuel Procyk

**Affiliations:** 1 Inserm, U846, Stem Cell and Brain Research Institute, Bron, France; 2 Université de Lyon, Lyon 1, UMR-S 846, Lyon, France; 3 Ernst Strüngmann Institute (ESI) in Cooperation with Max Planck Society, Frankfurt, Germany; Alexander Flemming Biomedical Sciences Research Center, Greece

## Abstract

**Background:**

It is increasingly recognized that non-motor symptoms are a prominent feature of Parkinson's disease and in the case of cognitive deficits can precede onset of the characteristic motor symptoms. Here, we examine in 4 monkeys chronically treated with low doses of the neurotoxin MPTP the early and long-term alterations of rest-activity rhythms in relationship to the appearance of motor and cognitive symptoms.

**Methodology/Principal Findings:**

Behavioral activity recordings as well as motor and cognitive assessments were carried out continuously and in parallel before, during and for several months following MPTP-treatment (12–56 weeks). Cognitive abilities were assessed using a task that is dependent on the functional integrity of the fronto-striatal axis. Rest-activity cycles were monitored continuously using infrared movement detectors of locomotor activity. Motor impairment was evaluated using standardized scales for primates. Results show that MPTP treatment led to an immediate alteration (within one week) of rest-activity cycles and cognitive deficits. Parkinsonian motor deficits only became apparent 3 to 5 weeks after initiating chronic MPTP administration. In three of the four animals studied, clinical scores returned to control levels 5–7 weeks following cessation of MPTP treatment. In contrast, both cognitive deficits and chronobiological alterations persisted for many months. Levodopa treatment led to an improvement of cognitive performance but did not affect rest-activity rhythms in the two cases tested.

**Conclusions/Significance:**

Present results show that i) changes in the rest activity cycles constituted early detectable consequences of MPTP treatment and, along with cognitive alterations, characterize the presymptomatic stage; ii) following motor recovery there is a long-term persistence of non-motor symptoms that could reflect differential underlying compensatory mechanisms in these domains; iii) the progressive MPTP-monkey model of presymptomatic ongoing parkinsonism offers possibilities for in-depth studies of early non-motor symptoms including sleep alterations and cognitive deficits.

## Introduction

Parkinson's syndrome (PS) was initially characterized by motor symptoms including rigidity, akinesia and tremor. However, it is increasingly recognized that these motor deficits are accompanied in 88% of PS patients by symptoms affecting cognitive performance, neuropsychiatric states, sleep, autonomic, and sensory domains which are important factors contributing to physical and mental disability [1], [2], [3], [4]. These non-motor symptoms have important consequences contributing to a decline in the quality of life. The causes of non-motor manifestations of PS could be multifactorial and related to widespread neurodegenerative processes of both dopaminergic and non-dopaminergic systems [5].

While numerous studies using animal models of Parkinson's disease (PD) have explored different aspects of these non-motor deficits, there is to date a lack of information concerning their overall orchestration with respect to the onset of classical clinical disease. We have therefore conducted a longitudinal study to simultaneously evaluate the temporal patterns of alterations in rest-activity cycles and cognition in the MPTP monkey model of PS.

The compensatory mechanisms and the concealed or partially concealed deficits that appear early on in PS, when the brain is undergoing major alterations during the pre-symptomatic period, are still not fully understood [6]. A long-standing literature has established that a low-dose MPTP regime in the monkey can induce long-lasting cognitive deficits resulting from fronto-striatal dysfunction, independent of the manifestation of any clinical signs of motor dysfunction [7], [8], [9], [10]. Other studies have shown that high doses of MPTP that rapidly engender severe clinical motor symptoms also influence biological rhythms and sleep [11]. However, it is not known if the impact of the dopaminergic lesion on biological rhythms occurs independently of either motor and/or cognitive effects during the so called pre-symptomatic period in which cognitive deficits are typically reported [12], [13].

While Parkinsonism is currently viewed as a complex network disorder [14], there is need for a more complete understanding of the spectrum of changes in PS that precede the diagnosis of classical motor deficits. This information is crucial for developing more effective and specific means of detecting disease onset as well as designing more effective therapeutics for the full range of symptoms. It follows that long term multi-dimensional survey of neurobiological states during the early stages of development of PS would be a valuable prerequisite for gaining a better understanding of the diagnostic features and timing of the different physiopathological components.

We therefore used continuous, long-term recordings in a non-human primate model of PS to analyze in parallel the changes in clinical scores, cognitive task performance and locomotor rest-activity patterns. We used chronic low-dose injections of MPTP to induce a Parkinson-like state that includes the development of compensatory mechanisms, thereby providing a model of low-grade dopaminergic lesion and sub-threshold clinical states [6]. Importantly, injections were suspended upon reaching a specific clinical threshold thereby favoring spontaneous motor recovery. However, analysis of behavioral parameters before, during and after chronic low-dose MPTP treatment demonstrated clear dissociations between clinical manifestations of motor dysfunction and both rest activity cycles and cognitive performances.

## Materials and Methods

### Ethics Statement

All procedures were carried out according to the 1986 European Community Council Directives (86/609/EEC), the French Ministère de l'Agriculture et de la Forêt, French Commission of animal experimentation, the Department of Veterinary Services (DDSV Lyon, France). These experiments were also carried out according to guidelines published in the Guide for the Care and Use of Laboratory Animals of the National Institutes of Health. Under local rules, authorization is granted to the laboratory for a range of experiments, rather than for specific studies. Specific authorization covering this study was delivered by the “Préfet de la Région Rhône Alpes” and the “Directeur départemental de la protection des populations” under Permit Number: #A690290402, including approved protocols in NHPs (#047, #048, #0198, #0199, #0200). All procedures complied with guidelines for animal welfare in accordance with the recommendations of the Weatherall report, “The use of non-human primates in research”. The nature of the measures and interventions described below required individual housing of monkeys during the experiment. This is also a welfare measure – symptomatic MPTP-treated monkeys would be at risk of persecution in a group-housing situation. Given this, however, all efforts were made to provide an enriched and social environment for the animals in a colony room with other familiar monkeys. All monkeys were closely monitored on a regular basis throughout the day, by several researchers as well as animal care staff, in order to ensure that levels of health and welfare were maintained. This was particularly the case during the MPTP period. As required, adaptations to housing and feeding procedures were made in direct response to individual symptoms in the MPTP phase, for example adaptations of water provision to ensure monkeys were able to drink *ad libitum* throughout.

### Animals and housing

Four monkeys were studied: three female *Macaca fascicularis* (monkeys J, F and L; aged respectively of 15, 12 and 11 years old) and a 13-year old male *Macaca mulatta* (monkey T), weighting 6, 5.5, 4.7 and 12kg, respectively. Animals were housed in a room dedicated to MPTP experiments, had free access to water and received food twice a day. Illumination was provided by white fluorescent tubes (Phillips Master, 15 Watts) mounted on the ceiling above the animal's cage providing 450–500 lux depending on the animal's position and gaze inside of the cage. The lighting schedule was clock-controlled for 14 hours light - 10 hours dark (14∶10 LD) for monkeys J and T and 12 hours light - 12 hours dark (12∶12 LD) for monkeys L and F. Mean room temperature during data collection was maintained at approximately 24–26°C.

### MPTP intoxication

A protocol of chronic low-doses (CLD) injections of 1-methyl-4-phenyl 1,2,3,6-tetrahydropyridine (MPTP) was employed based on previous studies [15] in order to obtain a slow onset of symptoms. CLD-MPTP treatment consisted of intramuscular injections of MPTP-HCl (Sigma M0896) diluted in sterile water at a concentration of 0.2 mg/kg. Injections were performed every 3 to 4 days in the home cage, without prior sedation. Pre-diluted doses of MPTP were prepared and stored in single-dose syringes (Myjector, Terumo®) at −80°C. The storage period did not exceed 2 months since MPTP can become unstable over longer periods [16]. NIH safety guidelines were respected for housing of treated-animals, MPTP preparation, storage, injection, and elimination. Injections of sterile water alone were performed previously to MPTP administration to ensure the absence of effects of injection procedure on measured behavioral parameters. As these animals were part of an ongoing study, they were not sacrificed at the end of the recordings and thus the extent of induced lesion on the aminergic system could not be verified. In one case (L), the levels of DA transporters was evaluated in-vivo in a follow-up PET study using a specific radioligand ([11C]-PE2I, [17]). Moreover, the cumulated doses used here (>2mg/kg) were superior to minimal cumulated dose used in previous studies that resulted in significant insult to the DA system [6], [18].

### Clinical Motor Evaluation

A clinical rating scale: the Parkinsonian Monkey Rating Scale (PMRS), adapted from the Unified Parkinson's Disease Scale (UPDRS) used for patients and compatible with rating scales used for monkeys [19], was employed to define the onset of the symptomatic period and to determine when to cease MPTP injections at the appropriate level of symptoms. We used the subtotal score of the PMRS corresponding to previously described scoring criteria in monkeys [20]: a score of 0 defined the asymptomatic stage; a score between 1 and 5 defined the pre-symptomatic period; a score above 5 defined the symptomatic stage. Once monkeys entered the symptomatic stage, injections were halted in order to gain information about possible spontaneous recovery. Clinical symptoms were assessed daily and the total score for freezing (0–3), resting tremor (0–3, for right and left side), rigidity (0–3), posture (0–2), bradykinesia (0–3), and ability to manipulate food (0–3, for right and left side) was used to frame injections (maximum total score of 23).

### Cognitive Behavioral task

Cognitive performance was monitored 5 days a week using a behavioral test previously employed to assess fronto-striatal functions and post-MPTP cognitive deficits [7], [8], [10]: Object Retrieval Detour Task (ORDT, **Figure S3**). The task was carried out in the animal's home cage and prior to injections during the MPTP period. Monkeys had to retrieve within a delay of 1 min a piece of fruit placed in a transparent box with an opening for access placed on only one side. Animals were tested for detour trials (see **Figure S3**) in which the open side was not facing the animal (either right or left side), requiring inhibition of the tendency to reach directly toward the reward and instead a strategy of a detour reach. However, each testing session contained a third of trials with the open side facing the animal so that the required reach was straight forward, in order to avoid automatic responses. Performance was evaluated by measuring successes and errors on detour trials. Successes (retrieval of reward on the first reach) were expressed as percentage of total number of trials. Errors (barrier hits i.e. hitting a transparent side of the box) were expressed as percentage of responses observed (there could be several responses per trial except in the case of success). A maximum of 18 trials per session were presented randomly to the animal each day. Animals initially received a short period of training to become accustomed to the presence of the experimenter and to learn the task; performance was then evaluated.

### Rest-wake activity profiles

Locomotor activity was recorded in the home cage (actimetry), continuously throughout the entire duration of the study (up to 16 months). Animal movements were detected using passive infrared captors placed above the cage and a monitoring system developed in the laboratory (Circadian Activity Monitoring System, CAMS, Inserm; see [21]). The captors detect the animal's displacements in the cage but are not sensitive to small tremor. Signals from the captors were recorded continuously and stored in 1 min bins (counts/min). The quantified activity thus represents spontaneous locomotor displacements throughout the rest-activity cycle. Monkeys were maintained in a 14∶10 or 12∶12 light: dark cycle. Maximum light intensity was 400 lux (Fluorescent white light 4000K) during the daytime period and 0 lux at night.

### L-Dopa treatment

A short-term dopatherapy was scheduled in cases J and T in order to investigate effects on: i) cognitive performances, and ii) circadian expression of locomotor rhythms post-MPTP. During the period of these tests (3–4 weeks), monkeys were treated twice-daily with oral administration of levodopa (Sinemet®, levodopa:carbidopa - ratio 10∶1) at a dose designed to produce a full reversal of the parkinsonian condition (15–20 mg.kg^−1^ of body weight; [22]). This was not carried out on cases L and F because they entered a cellular therapy protocol that will not be described here.

### Data analysis

We segmented MPTP and recovery periods into 4 equal epochs, 5 quantiles were then calculated from the beginning of each period using a 1-D linear interpolation method (MatLab interp1 function, de Boor, C. (1978) A Practical Guide to Splines. Springer-Verlag). These quantiles allowed a comparison of the time course of the variations of different parameters across an equivalent number of epochs for all subjects. This method of segmentation thus compares the stages of progression of processes linked to the aminergic lesion according to the behavioral outcomes rather than based on an absolute time-scale. Non-parametric circadian rhythm analysis (NPCRA) was used to extract actigraphic variables from diurnal activities [23]. These included measurements of (1) intra-daily stability (calculated as the variance of the average 24-hr pattern of activity around the mean and overall variance, indicating the consistency of day to day activity or the strength of coupling to the LD cycle i.e. the synchronizer) (2) intra-daily variability (indicating the frequency of transitions between rest and activity periods, corresponding to the fragmentation of activity) and (3) relative amplitude (ratio between the acrophase and the nadir of the rhythm, representing the ratio between the amplitude of activity in the light and dark phases). When the rest-activity cycle is stable, intra-daily stability is high, intra-daily variability is low (low fragmentation), and relative amplitude is high. Cognitive and clinical scores were also analyzed for the same quantile epochs using the same method to facilitate comparison between these three sets of scores.

### Statistics

Results are presented as means ± standard errors. Each monkey's weekly performance was compared with control by a treatment-contrast test using control measures as the first level (estimated standard errors and z-ratio were computed using GLM fit and contrast result was given by two-tailed p-values corresponding to z-ratio based on a Student t-test). Significance was considered for p<0.05. Statistical analyzes were computed using the R software (R Foundation for Statistical Computing, Vienna, Austrian http://www.R-project.org) and the summary function (Chambers, J. M. and Hastie, T. J. (1992) Statistical Models in S. Wadsworth & Brooks/Cole).

## Results

Clinical score, cognitive performance in a detour reaching task, and rest-activity cycles were followed continuously before, during and after MPTP-treatment for a period of up to 16 months in each of four macaque monkeys. While there was considerable variability in the sensitivity of individual animals to the CLD-MPTP treatment, the repeated sub-acute injections of MPTP reproduced non-motor symptoms typical of PS. The relative timing of the changes in rest activity and cognitive symptoms with respect to the onset of clinical motor symptoms were similar among all cases. In one case motor deficits persisted after MPTP treatment cessation and here cognitive and chronobiological deficits could not be dissociated. However, we observed long lasting non-motor symptoms in all cases studied and importantly in the three cases where there was recovery from motor deficits.

### Motor symptoms

**Figure 1:**
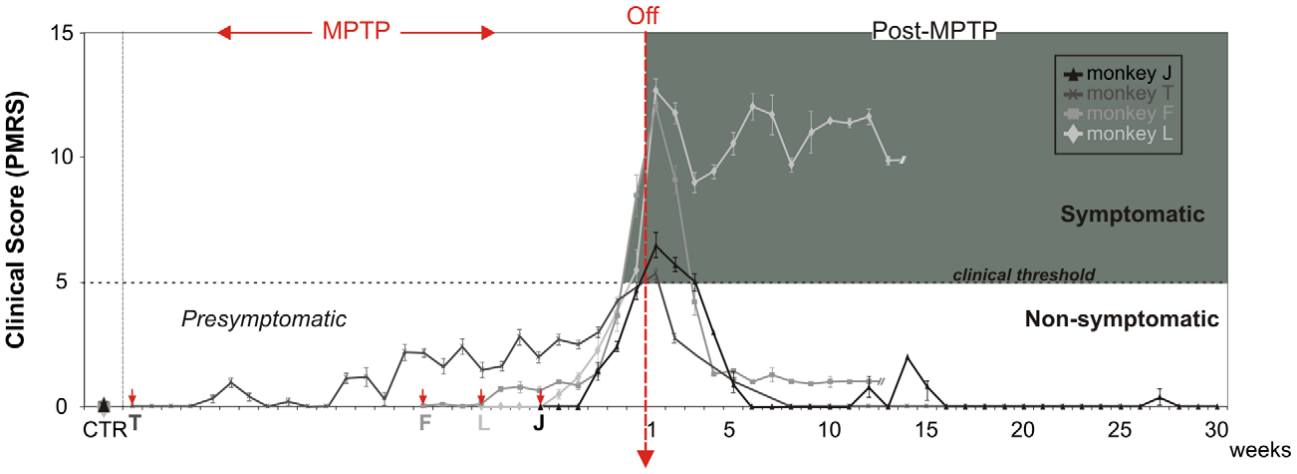
Progression of Clinical Scores in MPTP treated monkeys. Weekly clinical measures (mean ± SEM) for all monkeys aligned on MPTP treatment cessation (MPTP-Off). The shaded area corresponds to the symptomatic stage. During chronic low dose MPTP intoxication (MPTP) and prior to attaining the clinical threshold of 5, monkeys are considered as presymptomatic. After MPTP-Off, 3 monkeys spontaneously recovered to a non-symptomatic stage within 1–4 weeks while 1 monkey remained in a mild-symptomatic stage. Arrowheads in red indicate the time of MPTP onset for each animal.

**Figure 2:**
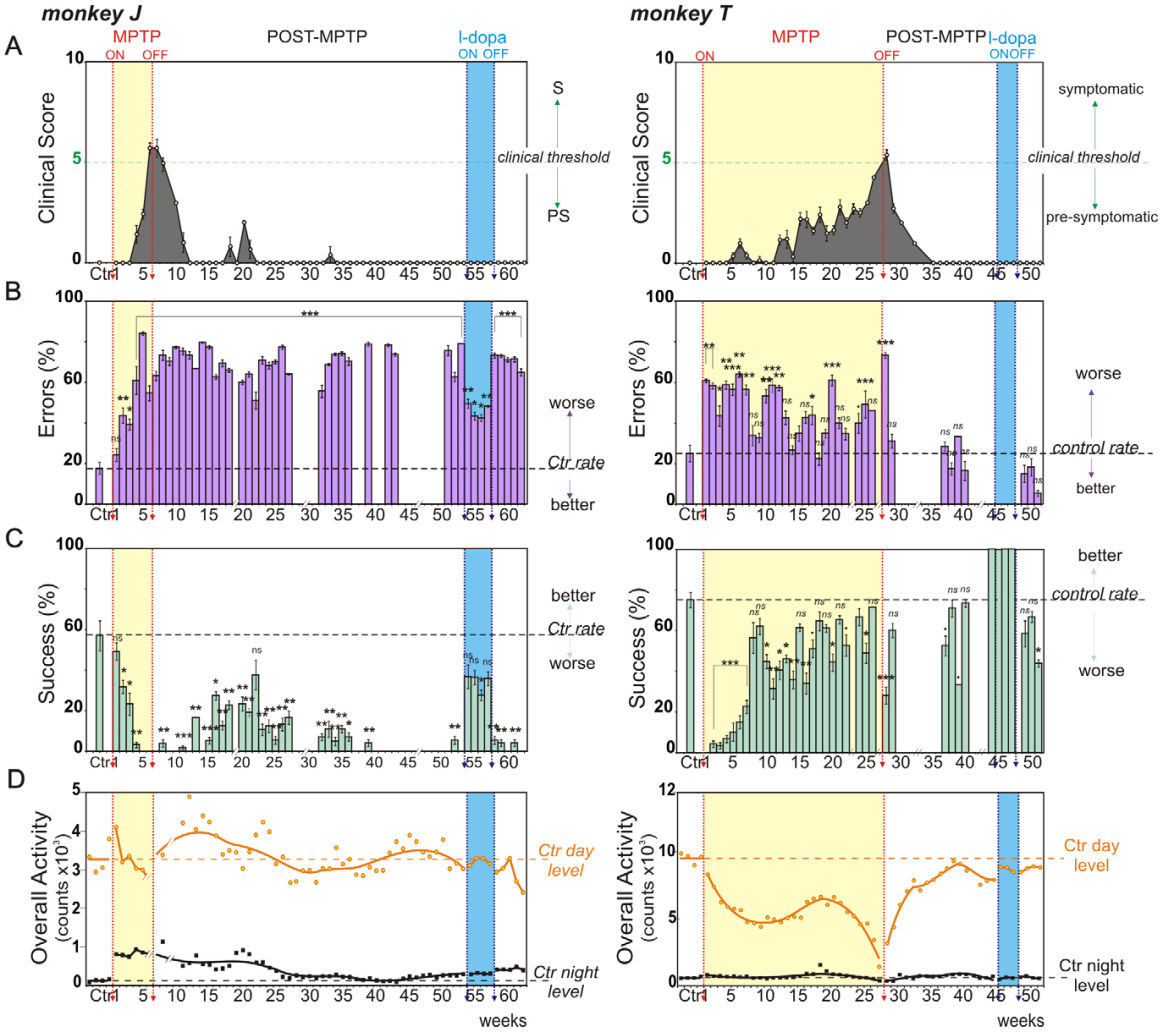
Evolution of behavioral parameters throughout the entire protocol. Weekly behavioral measures (mean ± SEM) for monkey J, left and monkey T, right. **A.** Clinical score variations from PMRS. Note that a score above 5 defines the symptomatic stage (clinical threshold, green line). **B.** Percentage of errors (barrier hits, purple) and **C.** percentage of successes (direct reach, blue) for the detour task (detour trials, see Figure S3). Performances (successes, errors) during and after MPTP-treatment are compared to control measures (horizontal dashed lines). **D.** Total daytime (orange circles) and night time (black squares) locomotor activity counts and associated curves of activity smoothed with the rloess method (span = 0.5). Baseline levels are represented by dotted lines for each behavioral parameter (Ctr day, Ctr night). Stars refer to statistics applied on Success and Error rates. ns: non-significant,**·**: p<0.1, *: p<0.05, **: p<0.01, ***: p<0.001.

**Figure 3:**
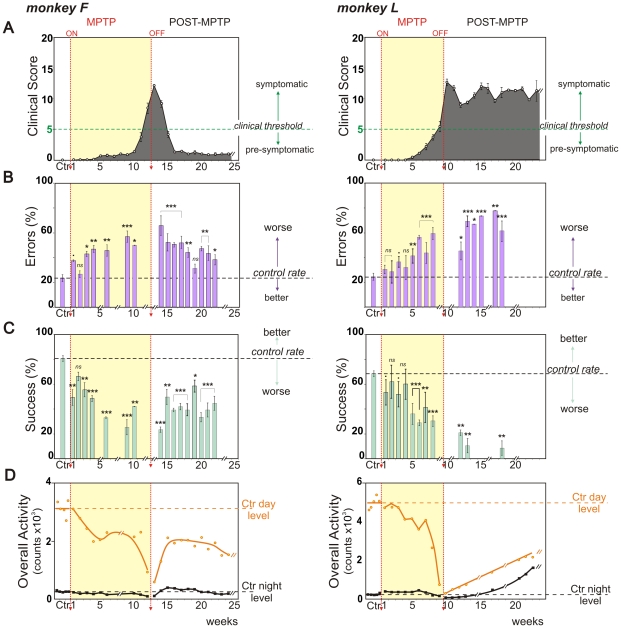
Changes of behavioral parameters over MPTP and Recovery periods. Weekly behavioral measures (mean ± SEM) for monkey F, left and monkey L, right. **A.** Clinical score variations from PMRS. **B.** Percentage of errors (barrier hits, purple) and **C.** Percentage of successes (direct reach, blue) at the detour task. **D.** Total daytime (orange circles) and night time (black squares) locomotor activity counts and associated curves of activity smoothed with the rloess method (span = 0.5). Conventions as for Figure 2.

All monkeys presented a clinical motor score of 0 in control conditions (Parkinsonian Monkey Rating Scale – PMRS, see materials and methods section) and after injections of sterile water. **Figure 1** represents changes in clinical scores of all cases studied aligned at the end of MPTP-treatment. Despite the different durations of MPTP intoxication required to attain clinical threshold and the different scores attained after MPTP-Off, the three monkeys that spontaneously recovered (cases J, T and F) presented relatively similar temporal dynamics of motor recovery (within 5–7 weeks). In case J the first clinical manifestation was observed 3 weeks after the onset of MPTP treatment and symptoms progressively increased up to 7 weeks when threshold (clinical score >5) was reached defining a pre-symptomatic period of 6 weeks, for a total cumulative dose of 2.2 mg/kg (**Figure 2A** **, left**). For case T, the onset of clinical symptoms occurred at 4 weeks and threshold was achieved at 28 weeks so that the pre-symptomatic period lasted considerably longer than in other cases (**Figure 2A** **, right**). In case T, we observed a marked but small peak in the clinical score at week 6 of treatment consisting of slight resting tremor (low amplitude and only intermittently present in the upper limbs). Given the weak response to MPTP in this case, doses were increased 2 fold for the last 3 weeks (generating a total cumulative dose of 10.4 mg/kg). In case F, clinical symptoms also appeared 5 weeks after the beginning of MPTP-treatment and remained stable and low (i.e. score<5) for the 4 following weeks (**Figure 3A** **, left**), here MPTP treatment was therefore increased from week 10 onwards, which led to a progressive increase of symptoms until week 12, when the clinical threshold was reached. The pre-symptomatic period in this case lasted 11 weeks for a total cumulative dose of 5.4 mg/kg. In case L, clinical symptoms emerged 5 weeks after initiation of treatment and increased continuously to reach threshold at week 9 (**Figure 3A** **, right**) for a total cumulative dose of 4.4 mg/kg and a pre-symptomatic period of 8 weeks, note that here the regimen of MPTP-injection was also increased in the last 3 weeks of intoxication.

Clinical symptoms emerged in the following order: action and resting tremor, bradykinesia, rigidity, stooped posture and freezing for cases J and L; action tremor, bradykinesia, stooped posture, resting tremor and freezing for case F; action tremor, bradykinesia, stooped posture, freezing and resting tremor for case T. The symptomatic period defined by a clinical score ≥5 lasted 17 days for case J, 24 days in case F and 6 days for case T. In case L, the symptomatic period lasted more than 12 weeks, this animal remained symptomatic after MPTP-Off with no spontaneous recovery. In this case, we confirmed that the lesion induced by MPTP progressed further than the critical threshold of 70–80% of striatal denervation [24], as shown by a separate study of DA transporter using PET-imaging with a specific radioligand ([11C]-PE2I, [17]). This showed that striatal denervation was >80% as estimated by binding potential calculation [25] two months after MPTP cessation (data not shown).

For the other cases (J, F and T) spontaneous recovery was consistently observed with clinical scores decreasing progressively after 1 to 5 weeks of symptomatic state (**Figure 2A** **,** **3A**). In case J some minor, transient bouts of motor symptoms were also observed around weeks 20 and 33 post-treatment. Following the last MPTP injection, recovery was observed in 6 weeks in case J, 5 weeks in case F and 7 weeks in case T.

Case J presented a persistent but slight action tremor of the upper limbs, which is not taken into account in PMRS scoring. This action tremor in case J was present throughout the pre-symptomatic period, stress enhanced and clearly visible during precise grasping movements. Levodopa treatment alleviated this condition by reducing action tremor amplitude and occurrence in the first week and making it rare to undetectable during the following weeks of therapy. It reappeared one week after levodopa offset. Case T presented a clinical score of 0 and no clear action tremor from week 35 onwards. In case T the only abnormality observed was a depressed-like behavior the week following levodopa offset (with little or no motivation to work for rewards). Case F presented a persistent slight bradykinesia throughout the rest of the protocol with a score of 1 (**Figure 3A** **, left**); L-Dopa was not tested in this case (see the material and methods, L-Dopa treatment section for details).

The clinical score variations for all cases when calculated according to quantile segmentation (see below), showed very similar sequential patterns over the intoxication and recovery periods, despite the individual differences in sensitivity to MPTP, in respect to both duration of effects and dose response.

### Cognitive Performances

Following one month of initial training, control performances in the detour reaching task (testing capacity to inhibit erroneous actions, see methods for further details) were averaged over the week preceding MPTP injections. Monkeys made relatively few errors and attained success rates of 60–80% (*Mean* ± *SD*; Case J: success rates 57±36%, error rates 17±15%; case T: success rates 69±25%, errors rates 30±23%; Case F: success rates 81±7%, error rates 23±8%; Case L: success rates 68±9%, error rates 24±10%; **Figure 2B,C** **and** **Figure 3B,C**).

For case J, MPTP performance was significantly worse than in the control conditions starting from the 2^nd^ week of injections (p<0.05, with Z-value<−2.4 for success and Z-value>2.3 error rates; **Figure 2B,C** **left**). The balance between error and success rates observed during the control period gradually reversed to an absence of success and an large increase of errors (more than 50%) at the end of MPTP intoxication (week 6). These poor performance levels, reminiscent of frontal-like behavior, remained constant even after the injections were terminated (p<0.05, Z-value<−2.1 for success rate and p<0.001, Z-value>3.4 for error rate; **Figure 2B,C** **left**).

For case T, performance in the first week of intoxication up to week 7 was significantly worse than during the control period (p<0.001, Z-value<−3.8 for success rate and p<0.05, Z-value>2.1 for error rate; **Figure 2B,C** **right**). During the remaining weeks of intoxication (week 8–27) performance continued to be globally worse than during the control period (**Figure 2B,C** **right**). After MPTP injections were terminated, case T recovered a control level of performance, except during the first week following MPTP-Off (p<0.01, with Z-value<−3.4 for success and Z-value>5.2 for error rates).

For case F, the first week of intoxication led to an abrupt decrease of performance and a return to control values in the 2^nd^ week (**Figure 3B,C** **left**). Subsequently performance deteriorated significantly with respect to the control condition and we observed again a reversal of the balance between success and errors. Success rate decreased and error rate increased progressively to reach maximum values at week 9 (p<0.001, with Z-value<−4.9 for success and Z-value>4.6 for errors). During the course of the MPTP-treatment, monkey F was sometimes reluctant to work for rewards and this was identified as a lack of motivation since generally monkey F would reach for fruit when offered outside the context of the task. Performance remained significantly worse than in the control condition for the entire period following MPTP-treatment cessation (12 weeks; p<0.05, with Z-value<−1.4 for success and Z-value>0.7 for errors; **Figure 3B,C** **left**).

For case L, performance deteriorated significantly from the 5^th^ week of injections (p<0.001, with Z-value<−3.5 for success and Z-value>2.7 for errors); but was noticeably altered beginning with the 1^st^ week of MPTP injections (**Figure 3B,C** **right**). In case L in the last weeks of MPTP-treatment the balance between success and errors was totally reversed (success rates 31±10%, error rates 59±12%). Because of a persistent, mild-parkinsonian state in case L, trial number per session for behavioral tests was reduced following MPTP-Off but was sufficient to show that performance remained constantly worse than in the control period (p<0.01, with Z-value<−2.1 for success and Z-value>1.9 for errors).

For case J, levodopa therapy improved performance (success rate >30%, error rate <50%; **Figure 2B,C** **left**) but did not allow recovery up to control levels. Performance for case T during levodopa therapy was better than during control (zero errors and 100% success rate throughout the 3 weeks of levodopa treatment; **Figure 2B,C** **right**).

Quantile comparison of error rates revealed similar dynamics across animals of the increasing difficulties to perform the task throughout the MPTP intoxication phase (see **Quantile Analysis section**). During the recovery period, these difficulties persisted for the three cynomolgus monkeys (error remained consistently higher than control levels for cases J, F and L) even though clinical scores had returned to non-symptomatic levels for cases J and F. However, for the rhesus (case T) the error rate rapidly returned to control levels.

### Rest-activity rhythms

**Figure 4:**
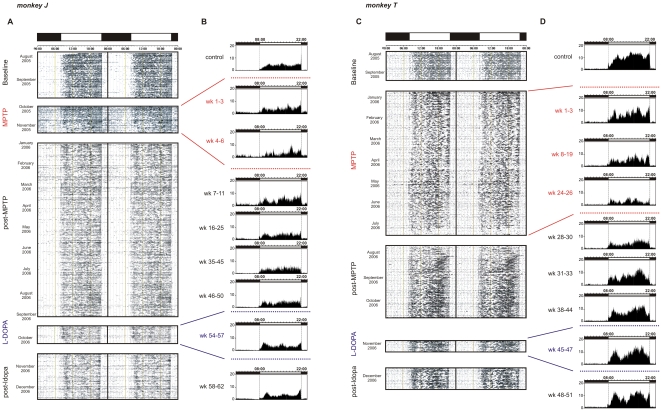
Locomotor activity rhythms (monkey J): A. 48h double-plot actogram: activity presented over consecutive days (each line is 48h) for the entire protocol. Daytime and night time periods are delimited by white and black rectangles, respectively (above actogram). Note the strong synchronization of activity at lights-off. **B.** 24h average locomotor activity profiles of 3 representative weeks taken over different time-windows of the protocol (time-window periods indicated on the left of profiles). Note the clear increase of night-time activity during MPTP and the progressive decrease after MPTP-Off. **Locomotor activity rhythms (monkey T): C.** 48h double-plot actogram and **D.** 24h average locomotor activity profiles (average over 3 representative weeks within the period indicated on the left). Note the clear fragmentation and amplitude decrease of daytime activity with MPTP intoxication and, the progressive return to near baseline after MPTP-Off.

During the initial control period all monkeys presented rest-activity rhythms that were highly structured and precisely synchronized with the light-dark (LD) cycle. Animals typically presented an immediate but progressive onset of activity at lights-on and an abrupt offset of activity at lights-off. Almost all activity (95.4±2%) was confined to the light phase (**Figure 4A,C** **and Figure S1A,C**). For case J, the mean±SD activity was 240.6±7.8 counts/hour during the day (light phase) and 12±0.6 counts/hour at night (dark phase); for case T, 617.4±17.4 counts/hour during the day and 48.6±1.2 counts/hour at night; for case F, 260±23.1 counts/hour during the day and 23.4±1.8 counts/hour at night and for case L, 413.9±25.6 counts/hour during the day and 18.3±3.5 counts/hour at night (**Figures 2D** **,** **3D**).

The rest-wake activity patterns during MPTP-intoxication are illustrated on the averaged profiles (**Figures 4B,D** **and Figure S1B,D**). All cases showed an abrupt and immediate change in rest-activity patterns from the first days of treatment, except in case L in which alterations were observed after one week of MPTP intoxication (**Figures 2D** **,** **3D**). In case J, these changes consisted of a sustained and abnormally elevated night-time activity throughout the period of MPTP treatment (**Figures 2D** **, left**). This was also the case for case L during the first 6 weeks of MPTP treatment (**Figures 3D** **, right**). For cases T, F and L, a progressive decrease in daytime activity was observed. The curves consistently showed a slight regain of daytime activity levels around the last third of the whole MPTP treatment period, just preceding a sharp decrease in activity that accompanied the invalidating clinical symptoms (**Figures 2A,D** **, and** **3A,D**). During the recovery period, rest-activity rhythms remained abnormally low for cases T, F and L for prolonged periods and failed to fully return to baseline levels throughout the entire time course of the observations (>11 months; **Figures 2D** **right, 3D, 4B,D and S1B,D**), long after spontaneous recovery from clinical symptoms. In case L, which failed to recover from motor symptoms, night time activity increased dramatically after MPTP-Off (**Figures 3D** **right, and S1C,D**).

In the two cases tested with levodopa (cases J and T), rest-activity rhythms were not affected in contrast to effects on cognitive performance (**Figures 2D** **and** **4A**–**D**).

### Quantile Analysis: Inter-individual comparison of behavioral changes

**Figure 5:**
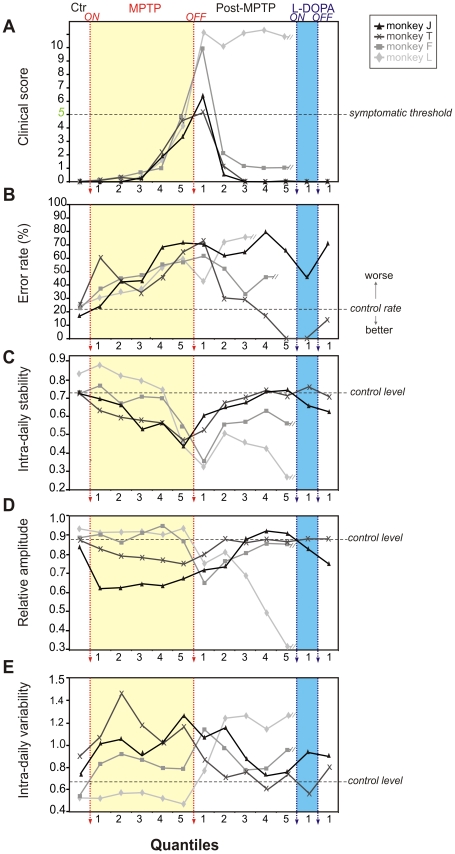
Quantile Analysis. Clinical Scores (**A**), behavioral performance (**B**, error rate) and measures on rest-activity rhythms representing intra-daily stability (**C**, strength of coupling), relative amplitude (**D**, rhythm intensity) and variability (**E**, fragmentation) for case J (black), case T (dark grey), case F (grey) and case L (light grey).

Since the four animals showed different vulnerabilities to the MPTP treatment resulting in large differences in the time required to display clinical motor symptoms (6, 8, 11 and 28 weeks), we analyzed several parameters according to subdivisions of time periods (quantiles) in order to disclose any similar trends in the appearance of the non-motor symptoms. This was assayed by dividing each phase (MPTP and post-MPTP) in an equal number of epochs. When normalized in this way, the evolution of the clinical scores during treatment and recovery exhibits similar patterns (**Figure 5A**), even though the time required to attain the critical clinical score of 5 differed significantly between animals (**Figure 1**). There was a similar trend in the increase of errors during treatment. However, during recovery cases J, L and F showed no post-treatment improvement while case T showed a sustained reduction in errors (**Figure 5B**).

All cases showed severely altered values for rest-activity parameters by the end of the first quantile of the treatment period, when clinical scores were still at baseline. Analysis of the intra-daily stability, the relative amplitude and the intra-daily variability of rest-wake activity revealed that all cases showed similar patterns of alteration during and after MPTP treatment (**Figure 5C**–**E**), with the exception of case L that showed more severe alterations. Intra-daily stability of the rest-activity cycle (representing the strength of coupling of the rest-activity cycle to the external light-dark cycle [26]) decreased immediately after initiation of MPTP treatment, and continued to decrease throughout treatment. For cases J and T stability only returned to baseline levels several months after MPTP treatment ended while in cases F and L there was no recovery (case J 8 months and case T 3 months, **Figure 5C**). The relative amplitude represents the strength of the rhythmic oscillation of motor activity and is obtained by comparing the difference between the most and least active periods. Relative amplitude was also found to evolve in a similar manner during recovery for the 3 monkeys (J, T, F; **Figure 5D**) only returning to baseline levels months after treatment (and not at all for monkey L). Intra-daily variability, a parameter that reflects the fragmentation of activity (i.e. frequency and extent of transitions between activity and rest) consistently increased during and after MPTP treatment (**Figure 5E**). Thus, this method of comparison showed similar outcomes during and after MPTP treatment for the spontaneously recovered cases. For case L, that continued in a symptomatic state, all parameters remained dramatically affected.

## Discussion

We have designed a protocol for chronic multidimensional characterisation of non-motor aspects of PS. Despite inter-individual differences, monitoring a range of behavioural parameters, our results clearly show that CLD-MPTP induces early and long-term non-motor features of idiopathic PS (see Table S1 for a summary of main results). Furthermore, all cases exhibited very early changes in both rest-activity cycles and cognitive performances, virtually immediately after the first MPTP administration and well before clinical motor symptoms.

A key finding is the demonstration that the MPTP induced changes in chronobiological parameters constitute a precocious and consistent feature of the pre-symptomatic phase that precedes or coincides with measurable cognitive decline (i.e. after the 1^st^ MPTP injection in case J and L, the second MPTP injection in case T and the 6^th^ injection in case F). Identification of the temporal sequence of behavioral events (chronobiology, cognitive, motor) subsequent to MPTP intoxication benefited from several factors. The within subject experimental design allowed each animal to serve as its own control, decreasing variability and increasing the sensitivity of the measures. The long-term, continuous 24h monitoring of behavior permitted a fine grain analysis of behavioral decline for several chronobiological parameters.

### Cognitive deficits

The cognitive decline induced by MPTP-intoxication was typically pre-symptomatic (i.e. before the onset of characteristic parkinsonian motor symptoms) and persisted after full clinical recovery. The detour task exploited here is very simple to learn, easy-to-test, has been used in numerous protocols and is considered to be dependent on the integrity of frontal cortex, the dopaminergic system and DA innervation of frontal cortex. For example, these tests were used by Diamond and Goldman-Rakic in the 1980′s to demonstrate that human infants and infant monkeys show a clear developmental progression of cognitive abilities that accompanies maturation of the DA system (see [27] for a review). Cognitive operations, crucial for planning and problem solving, are known to be impaired in both parkinsonian patients and CLD-MPTP monkeys [9], [10], [28], [29]. In monkeys, cognitive and complex motor planning abilities following MPTP treatment have previously been tested using various protocols including extra- and intra-dimensional shifting tasks, delayed response tasks, Go No-Go tasks, context dependant movement selection (automated version of the ODRT), multiple choice retrieval tasks, and the object detour retrieval task [9], [10], [13], [30]. These investigations have shown that cognitive impairments can occur without clinical symptoms when using low-dose MPTP injections with, in some cases, maintenance of deficits several weeks (e.g. [31]) or years after MPTP exposure [32]. Here we show that the detour task revealed deficits within one to five weeks of low dose treatment. Previous investigations also reported cognitive deficits at various delays depending on the injection protocol [10], [13], [30]. Inter-individual variability of the onset is frequently reported. Our day by day examination of cognitive performance showed the first significant decline to be detectable after 1^st^ injection at day 2 in case T, 4^th^ injection at day 11 in case J, 2^nd^ injection at day 4 in case F and after the 5^th^ injection at day 19 in case L.

The classical DA levodopa therapy protocol clearly improved performance in cases J and T. In case J the error rate decreased and the success rate improved to levels that were not significantly different to those prior to MPTP treatment. In case T, DA levodopa therapy led to 100% success rates and zero failure rates, rates that were actually superior to those encountered prior to MPTP treatment. These results indicate that poor cognitive performances can be imputed to a dopaminergic deficit in the brain.

Our results thus confirm previous observations and indicate that the detour task allows detection of early presymptomatic frontal behavior deficits during CLD-MPTP and their possible maintenance after clinical recovery.

### Chronobiology

Following the MPTP treatment and despite a recovery from motor symptoms, the rest-activity profiles did not return to a completely normal state throughout the duration of the experimental observation period. These findings differ from previous studies of daily activity patterns in MPTP monkeys that mainly showed (i) a decrease in the overall amount of activity without any obvious modification of the day-night activity profiles and (ii) changes in rest-activity uniquely when clear motor symptoms were present [33]. This latter finding differs from our results where changes in activity profiles are found in the presymptomatic stage. These differences with our results may be due to the mode of intoxication (i.e. intravenous injections of MPTP with twice the concentration compared to what we used), the age of monkey used (3 years old i.e. juvenile) and the lack of pre-intoxication control measures from the same monkey. Intravenous MPTP-treatment, corresponding to an acute intoxication is known to be much more offensive towards the DA system and induces a different topography of the DA lesion within basal ganglia [34]. It has also been shown that older monkeys are more suitable to reproduce the characteristic features of this age-related disease [35], [36]. Our results are consistent with those of Barraud et al. (2009) [11] that showed a long-term disruption of sleep–wake architecture and a reduced sleep efficiency that can be observed years after MPTP administration in monkeys suffering from chronic clinical motor symptoms following MPTP administration.

The present findings extend the observations of others by showing that alterations of the locomotor activity rhythm can persist in the absence of stable clinical symptoms and are not necessarily correlated with the degree of motor disability [11], [33]. The persistence of activity alteration is particularly evident in the quantile analyses. The quantile analysis allows a representation of the behavioral effects during the different periods according to the experimental stages rather than on their durations and provides a means to equate the behavioral changes independent of the time required to attain the clinical criteria. This analysis shows that despite the different effects on day and night time activities following motor recovery, all cases presented similar dynamics in the deterioration of the stability, amplitude and variability of the rest-wake cycle.

Alterations in sleep, including excessive daytime sleepiness, sleep fragmentation and rapid eye movement (REM) sleep deregulation are observed in MPTP treated monkeys [11] and in PD patients during early and symptomatic stages of the disease [37]. In humans, actigraphy is routinely used to detect circadian rhythm disorders [38]. The technique has also been validated against polysomnography to assess the quality and fragmentation of sleep and wakefulness [39], [40]. Although in monkeys actigraphy has not been compared to polysomnography, it is likely that, as in humans, an increase in motor activity during the night time is a marker of sleep disturbance, and that a decrease in activity during the daytime could be a sign of daytime sleepiness, in agreement with Barraud et al. (2009) [11]. These two changes in activity that we observed during both the presymptomatic and symptomatic phases, could therefore mimic the patterns of sleep fragmentation and excessive daytime sleepiness observed in PD patients [41].

The origin of these rest-activity (or sleep) disturbances in MPTP monkeys is still a matter of speculation. In MPTP treated monkeys some studies e.g. [42] have reported loss of dopaminergic as well as other monoaminergic cell populations (noradrenalin, serotonin) whereas others report only dopamine degeneration e.g. [11] or together with increased levels of serotonin [43]. PD patients show multiple neuronal system degenerations [44]. Furthermore, it is unknown whether the observed chronobiological alterations can be attributed to direct or indirect consequences of a breakdown of dopamine homeostatic regulation affecting the sleep-wake system and/or the circadian oscillator system. At least some outputs of the circadian control system appear to be conserved, since the concentration and rhythms of melatonin, cortisol and prolactin secretion reportedly remain largely intact [45].

### Inter-individual variability

The four monkeys showed a differential vulnerability to the MPTP treatment, in agreement with previous studies [6], [7], [18]. All three female cynomolgus showed highly similar early and long-lasting non-motor alterations during and after CLD-MPTP treatment. Despite inter-individual differences we clearly show that all cases exhibited very early changes in both rest-activity cycles and cognitive performances, virtually immediately after the firsts MPTP administration and prior to clinical motor symptoms.

In case T the period of intoxication required to reach clinical significance was nearly 3 to 5 times longer than for other cases. The lower sensitivity of case T to MPTP at the doses and frequency employed here (0.2 mg.kg^−1^ every 3–4 days) may have allowed specific compensatory mechanisms that were distinct from those in other cases, which however did not differentially affect the expression of non-motor symptoms (here rest-activity cycle alterations and cognitive deficits). In case T, cognitive difficulties in the detour reaching task were stable during the MPTP treatment but were practically undetectable after spontaneous clinical recovery. We observed that case T learned the detour reaching task more rapidly than other cases. In view of these observations case T was also trained to a more complex delayed response task (spatial working memory task with a delay of 10sec, **Figure S2**) that revealed stable cognitive deficits throughout the presymptomatic period and persistence after full clinical recovery. These results are in agreement with those showing that the level of cognitive deficits depends on task difficulty, in both parkinsonian patients [46] and rhesus monkeys treated with MPTP [13].The variability between subjects could originate from various factors ranging from individual genetic susceptibilities to the treatment, age, species or gender differences [6], [18], [47]. Although several species (macaques, vervet monkeys) and both genders have been used in previous experiments no comprehensive comparative studies have been made with non-human primates. The issue of variability is also illustrated by the fact that fully clinically recovered animals can present DA cell loss that is equivalent to those observed for severe symptomatic cases [6]. Similarly, asymptomatic-recovered monkeys show similar changes in subcortical/cortical aminergic levels compared to those observed for symptomatic cases [48].

Here we show that the use of quantile analyses to equate the different durations of each phase overcomes some aspects of individual variability and allows a more rationalized behavioral evaluation. Nevertheless the inter-variability observed in response to treatment or for post-treatment recovery in the MPTP model is clearly of value since it may help to better understand the variability of human PS.

### Compensatory mechanisms

The use of CLD-MPTP models potentially allows determination of the functional basis of the compensatory mechanisms leading to or accompanying spontaneous motor recovery and possibly involving more than one aminergic system [6], [42], [49]. Our finding that chronobiological and cognitive symptoms persist in motor recovered animals poses a number of questions concerning the physiological status of motor-recovered animals. Along with previous studies that have shown cognitive deficits during the presymptomatic period, the present findings emphasize that the full spectrum of deficits during this stage is more extensive than previously thought. This kind of protocol (longitudinal and using the CLD-MPTP model) aims at reaching deeper insights into the presymptomatic period and should be further used to explore if neurophysiological markers of sleep structure [11] or of performance monitoring [50] can ultimately be used as reliable diagnostic tools for ongoing PS in humans.

## Supporting Information

Figure S1 **Locomotor activity rhythms (monkey F): A.** 48h double-plot actogram: activity presented over consecutive days (each line is 48h) for the entire protocol. Daytime and night time periods are delimited by white and black rectangles, respectively (above actogram). Note the strong synchronization of activity at lights-off and fragmentation of daytime activity with MPTP treatment. **B.** 24h average locomotor activity profiles of 3 representative weeks taken over different time-windows of the protocol (week periods indicated on the left of profiles). **Locomotor activity rhythms (monkey L): C.** 48h double-plot actogram and **D.** 24h average locomotor activity profiles (average over 3 representative weeks within the period indicated on the left). Note the clear fragmentation and amplitude decrease of daytime activity with MPTP intoxication. While we can observe a progressive return of daytime activity after MPTP-Off, this is far from baseline levels and moreover accompanied by a strong increase in night time activity.
(TIF)

Figure S2 **Spatial memory task. A.** Spatial delayed retrieval task paradigm. The monkey is facing 3 wells with opaque cover; first, wells are opened so that the monkey can see which one contains the reward. Then an opaque screen is placed between animal and wells during 10sec; after the delay the monkey had to reach the correct well to obtain the reward. In case of error, the trial start again until reward is found. Perseverance in incorrect choices is carefully noted: incorrect trials for which the monkey makes the same incorrect choice as in the previous trial. This task evaluates the integrity of short term spatial memory. **B.** Clinical score (up) and performances (down) of monkey T along the entire protocol. Note the clear expression of frontal-like behavior (perseverance, black bars) with MPTP intoxication and persistence after MPTP-off. *In* ***B.*** stars correspond to statistical results of contrast with control values (see material and method section); *: p<0.05, **: p<0.01, ***^.^*** *: p<0.07 i.e. marginally significant.*
(TIF)

Figure S3 **Detour Task paradigm.** In this task monkeys have to reach a piece of fruit placed in a transparent box with only one opened window. In direct trials (left), the opening is facing the animal so that reach is straightforward; in detour trials (right), the opening is not facing the monkey so that a detour has to be planned before reaching the reward. This easy-to-learn and easy-to-test task evaluates lack of inhibition, behavior dependent on the integrity of frontal cortex, the dopaminergic system and DA innervation of frontal cortex.
(TIF)

Table S1 **Summary table of results.** Behavioral data and MPTP cumulated dose are shown for each monkey (columns) and for the major periods of the follow-up study: control (CTR), presymptomatic state (PMRS score <5), symptomatic state (PMRS score >5) and recovery period. -: not available.
(DOCX)
