# Supplementary figures and images for: Early Presymptomatic and Long-Term Changes of Rest Activity Cycles and Cognitive Behavior in a MPTP-Monkey Model of Parkinson's Disease
Source: PLoS One. 2011 Aug 24;6(8):e23952. doi: 10.1371/journal.pone.0023952 (PMC3161087; doi:10.1371/journal.pone.0023952)

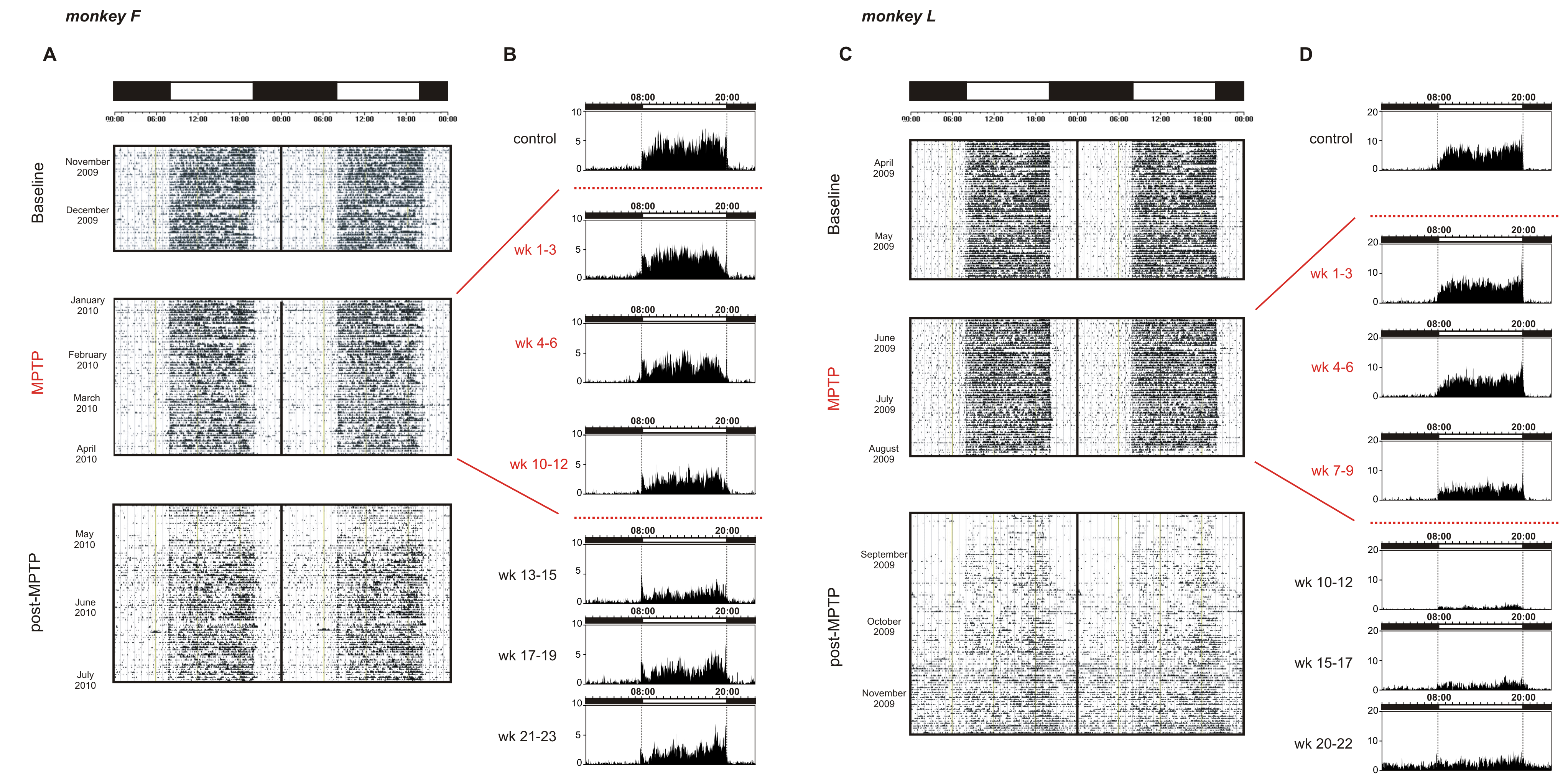

Supplement: Figure S1 — Locomotor activity rhythms (monkey F): A. 48h double-plot actogram: activity presented over consecutive days (each line is 48h) for the entire protocol. Daytime and night time periods are delimited by white and black rectangles, respectively (above actogram). Note the strong synchronization of activity at lights-off and fragmentation of daytime activity with MPTP treatment. B. 24h average locomotor activity profiles of 3 representative weeks taken over different time-windows of the protocol (week periods indicated on the left of profiles). Locomotor activity rhythms (monkey L): C. 48h double-plot actogram and D. 24h average locomotor activity profiles (average over 3 representative weeks within the period indicated on the left). Note the clear fragmentation and amplitude decrease of daytime activity with MPTP intoxication. While we can observe a progressive return of daytime activity after MPTP-Off, this is far from baseline levels and moreover accompanied by a strong increase in night time activity. (TIF) [file pone.0023952.s001.tif]

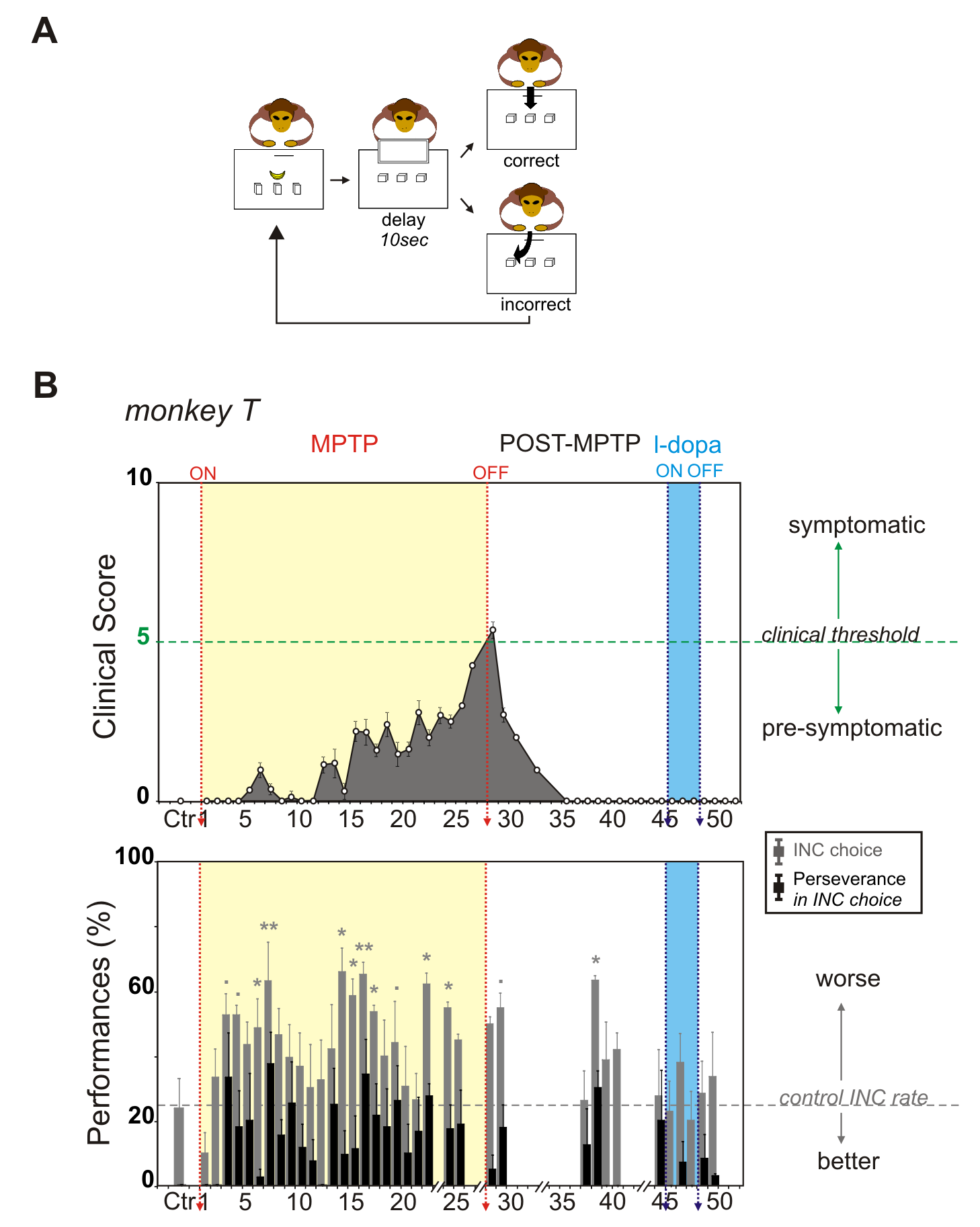

Supplement: Figure S2 — Spatial memory task. A. Spatial delayed retrieval task paradigm. The monkey is facing 3 wells with opaque cover; first, wells are opened so that the monkey can see which one contains the reward. Then an opaque screen is placed between animal and wells during 10sec; after the delay the monkey had to reach the correct well to obtain the reward. In case of error, the trial start again until reward is found. Perseverance in incorrect choices is carefully noted: incorrect trials for which the monkey makes the same incorrect choice as in the previous trial. This task evaluates the integrity of short term spatial memory. B. Clinical score (up) and performances (down) of monkey T along the entire protocol. Note the clear expression of frontal-like behavior (perseverance, black bars) with MPTP intoxication and persistence after MPTP-off. In B. stars correspond to statistical results of contrast with control values (see material and method section); *: p<0.05, **: p<0.01, . : p<0.07 i.e. marginally significant. (TIF) [file pone.0023952.s002.tif]

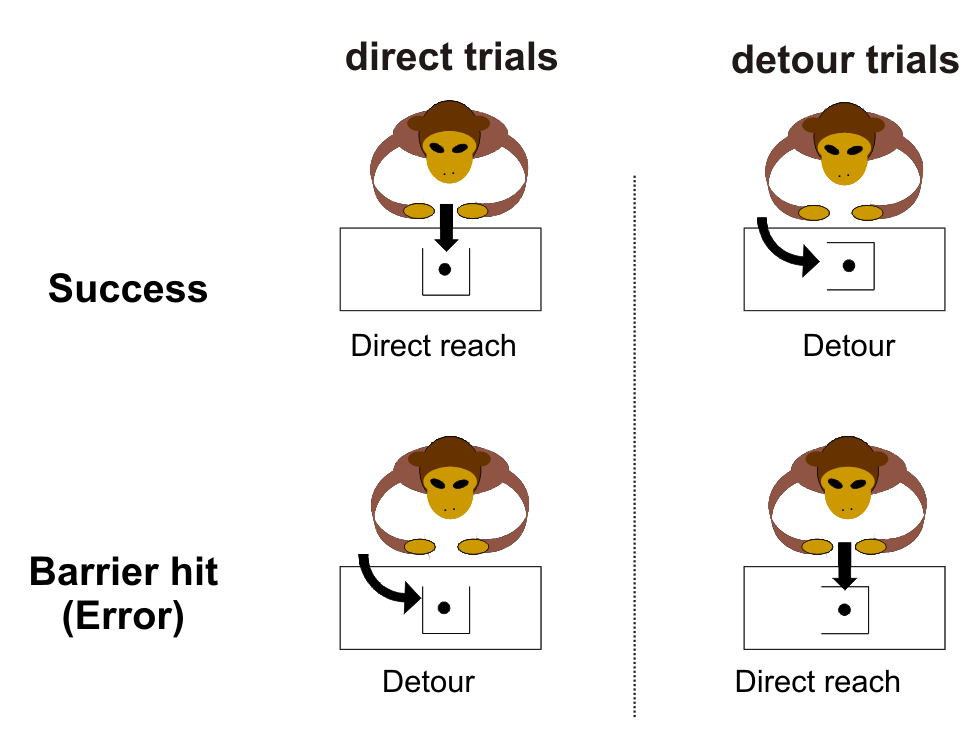

Supplement: Figure S3 — Detour Task paradigm. In this task monkeys have to reach a piece of fruit placed in a transparent box with only one opened window. In direct trials (left), the opening is facing the animal so that reach is straightforward; in detour trials (right), the opening is not facing the monkey so that a detour has to be planned before reaching the reward. This easy-to-learn and easy-to-test task evaluates lack of inhibition, behavior dependent on the integrity of frontal cortex, the dopaminergic system and DA innervation of frontal cortex. (TIF) [file pone.0023952.s003.tif]
