# Supplementary material for: Early Presymptomatic and Long-Term Changes of Rest Activity Cycles and Cognitive Behavior in a MPTP-Monkey Model of Parkinson's Disease
Source: PLoS One. 2011 Aug 24;6(8):e23952. doi: 10.1371/journal.pone.0023952 (PMC3161087; doi:10.1371/journal.pone.0023952)
Supplement: Table S1 — Summary table of results. Behavioral data and MPTP cumulated dose are shown for each monkey (columns) and for the major periods of the follow-up study: control (CTR), presymptomatic state (PMRS score <5), symptomatic state (PMRS score >5) and recovery period. -: not available. (DOCX) [file pone.0023952.s004.docx]

|  | **monkey J** | **monkey T** | **monkey F** | **monkey L** |
| --- | --- | --- | --- | --- |
| **Duration (in weeks)** |  |  |  |  |
| **Presymptomatic state** | **6** | **27** | **11** | **8** |
| **Symptomatic state** | **2** | **1** | **3** | **>12** |
| **Recovery period** | **6** | **7** | **5** | **-** |
| **Total cumulated dose (in mg/kg)** | **2.2** | **10.4** | **5.4** | **4.4** |
| **ORDT (errors %, mean ±SD)** |  |  |  |  |
| ***CTR*** | ***17 ±15*** | ***25 ±20*** | ***23 ±8*** | ***24 ±10*** |
| **Presymptomatic state** | **51 ±21** | **46 ±12** | **43 ±10** | **41 ±12** |
| **Symptomatic state** | **68 ±7** | **73 ±7** | **65 ±18** | **66 ±11** |
| **Recovery period** | **74 ±3** | **31 ±17** | **51 ±0.7** | **-** |
| **ORDT (success %, mean ±SD)** |  |  |  |  |
| ***CTR*** | ***57 ±36*** | ***75 ±19*** | ***81 ±7*** | ***68 ±9*** |
| **Presymptomatic state** | **18 ±20** | **40 ±23** | **46 ±14** | **46 ±13** |
| **Symptomatic state** | **2 ±3** | **28 ±21** | **23 ±6** | **7 ±8** |
| **Recovery period** | **0.5 ±1** | **60 ±17** | **44 ±6** | **-** |
| **Overall activity, *average counts x10^3^*** |  |  |  |  |
| **(Day/night counts, in % of Total)** |  |  |  |  |
| ***CTR*** | ***3.3/0.1 (97/3)*** | ***7.9/0.5 (94/6)*** | ***3.1/0.3 (92/8)*** | ***4.9/0.2 (96/4)*** |
| **Presymptomatic state** | **3.3/0.8 (81/19)** | **4.5/0.5 (90/10)** | **2.4/0.2 (92/8)** | **4.1/0.3 (92/8)** |
| **Symptomatic state** | **3.4/1.1 (75/25)** | **2.7/0.3 (91/9)** | **1.0/0.2 (84/16)** | **1.2/0.5 (69/31)** |
| **Recovery period** | **4.6/0.5 (90/10)** | **5.7/0.4 (93/7)** | **2.0/0.4 (84/16)** | **-** |
